# Late Pleistocene osseous projectile point from the Manis site, Washington—Mastodon hunting in the Pacific Northwest 13,900 years ago

**DOI:** 10.1126/sciadv.ade9068

**Published:** 2023-02-01

**Authors:** Michael R. Waters, Zachary A. Newell, Daniel C. Fisher, H. Gregory McDonald, Jiwan Han, Michael Moreno, Andrew Robbins

**Affiliations:** ^1^ Center for the Study of the First Americans, Department of Anthropology, Texas A&M University, College Station, TX 77843, USA.; ^2^ Department of Anthropology, Oregon State University, Corvallis, OR 97331, USA.; ^3^ Museum of Paleontology and Department of Earth and Environmental Sciences, University of Michigan, 1105 North University Ave., Ann Arbor, MI 48109-1085, USA.; ^4^ Bureau of Land Management, Utah State Office, West 200 South, Salt Lake City, UT 84101, USA.; ^5^ J. Mike Walker ‘66 Department of Mechanical Engineering, Texas A&M University, College Station, TX 77843, USA.; ^6^ Multidisciplinary Engineering Department, Texas A&M University, College Station, TX 77843, USA.

## Abstract

Bone fragments embedded in a rib of a mastodon (*Mammut americanum*) from the Manis site, Washington, were digitally excavated and refit to reconstruct an object that is thin and broad, has smooth, shaped faces that converge to sharp lateral edges, and has a plano-convex cross section. These characteristics are consistent with the object being a human-made projectile point. The 13,900-year-old Manis projectile point is morphologically different from later cylindrical osseous points of the 13,000-year-old Clovis complex. The Manis point, which is made of mastodon bone, shows that people predating Clovis made and used osseous weapons to hunt megafauna in the Pacific Northwest during the Bølling-Allerød.

## Teaser

Bone projectile points were used to hunt mastodon at the end of the last Ice Age.

## INTRODUCTION

The only prehistoric weapon demonstrated to have been used to hunt North American megafauna during the late Pleistocene south of the Laurentide and Cordilleran Ice Sheets is the Clovis projectile point (*1*, *2*). These points were made of stone, date from ~13,050 to ~12,750 calibrated years before the present (cal yr B.P.), and are found to be associated with mammoths, mastodons, and gomphotheres (*3*, *4*). Stone projectile points predating Clovis have been found at North American sites, but none are directly associated with megafaunal carcasses (*5*–*8*). Conversely, megafauna kill or scavenging sites predating Clovis have been found with associated lithic tools but with no associated projectile points (*9*–*11*). At the Manis site in northwest Washington, a fragmented bone object is embedded in a rib of a mastodon (*12*). Here, we show that these fragments are from a bone projectile point that was used to hunt a mastodon in the Pacific Northwest ~13,900 years ago, some 900 years before Clovis.

**Fig. 1.**
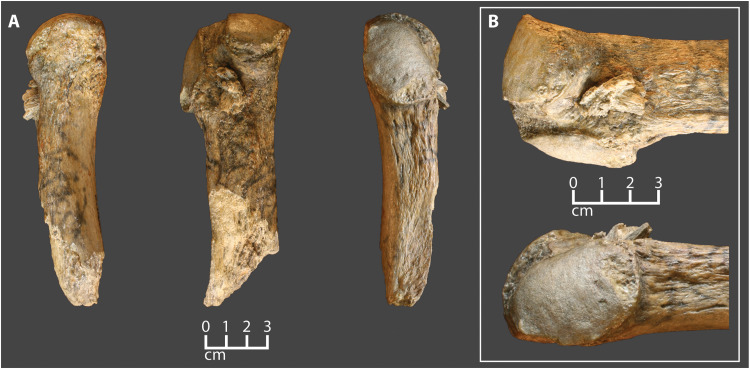
The Manis mastodon rib with embedded projectile point. (**A**) Three views of the rib fragment with embedded point. (**B**) Close-up views of the embedded point. Note root staining on the bone and embedded bone point.

At the Manis site, a single male mastodon (*Mammut americanum*) was excavated from sediments at the base of a kettle pond by Carl Gustafson from 1977 to 1979 (*12*, *13*). No stone tools were found at the site, but human interaction with the mastodon was suggested by bones with spiral fractures, flakes removed from a long bone, and bones with cut marks (*12*, *14*, *15*). In addition to this, the proximal end of a right rib had foreign osseous fragments embedded in it (Fig. 1). These foreign pieces were interpreted to be the tip of an osseous projectile point that broke apart as it impacted and entered the rib (*12*, *13*). This rib with its inferred projectile point fragments were recovered ex situ from sediments excavated by the backhoe that first exposed the mastodon skeleton. Organic matter associated with the in situ bones provided an original age estimate of ~14,000 cal yr B.P. However, this age and the human origin of the osseous material in the rib were challenged, and the evidence for pre–13,000-year-old human-mastodon interaction at the Manis site was treated as equivocal (*2*).

In 2011, an anatomical study showed that the rib with the embedded bone fragments was the 14th right rib. High-resolution x-ray computed tomography (CT) revealed that the osseous object embedded in the rib was dense bone that fragmented into multiple pieces upon impact as it penetrated 2.15 cm into the rib (*16*). DNA analysis of the intrusive osseous fragments showed that they were from a mastodon (*16*). Four accelerator mass spectrometry radiocarbon ages on XAD-purified bone collagen extracted from the mastodon rib containing the embedded osseous pieces and from both tusks showed that the Manis mastodon dated to 11,960 ± 17 radiocarbon years before the present (*16*). The IntCal20 radiocarbon calibration (*17*) revises this age to 13,790 to 13,995 cal yr B.P. or ~13,900 cal yr B.P. Despite this evidence, the interpretation that the embedded osseous pieces in the rib represented the fragmented tip of a projectile point was again challenged, and three alternative explanations were proposed to refute the hypothesized prehistoric interaction of humans with the Manis mastodon [see the Supplementary Materials; (*3*, *18*–*20*)].

**Fig. 2.**
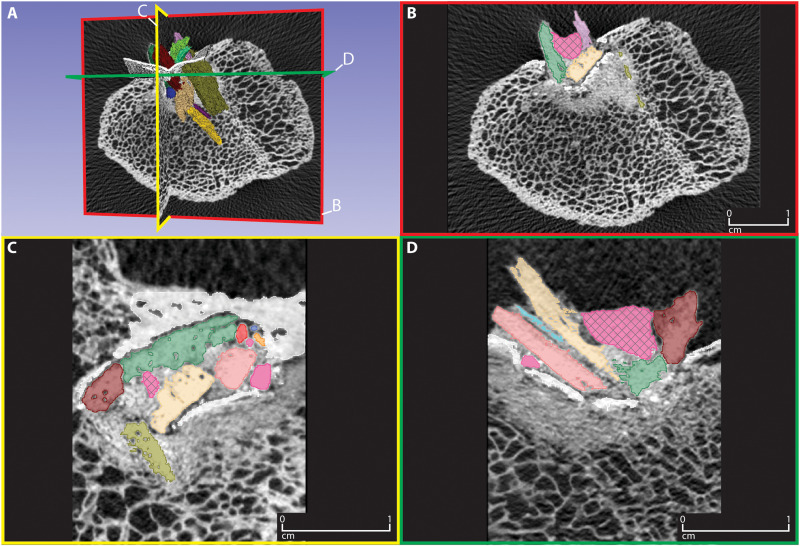
Micro-CT images of the Manis rib. These images show the exterior cortical bone (white, representing high x-ray attenuation), interior trabecular bone (gray, representing lower x-ray attenuation), and the projectile point fragments (colored for identification, but originally white, like other high-attenuation materials). (**A**) Embedded point fragments in color showing the intersecting transverse, axial, and horizontal planes depicted in (B) to (D). (**B**) Transverse plane (red). (**C**) Axial plane (yellow). (**D**) Horizontal plane (green). Scale in images (B) to (D). The dark pink mass with a cross hatch pattern is the putty used to fill the hole created during DNA sampling and stabilize the surface bone. The small dark pink pieces without cross hatching are small fragments that were not used in the reconstruction.

**Fig. 3.**
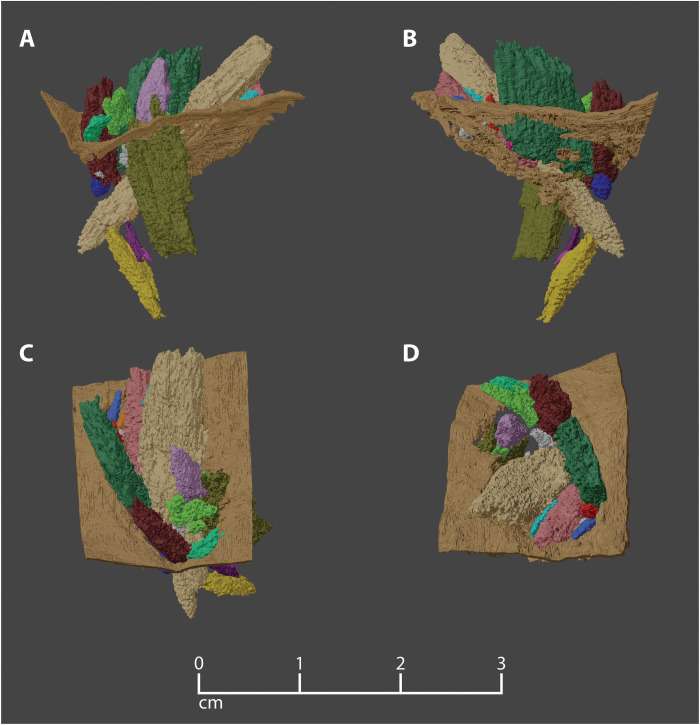
Projectile fragment cluster embedded in the Manis rib. Multiple views of cluster of projectile fragments within the wound in the Manis rib. Cortical bone (brown) trimmed to show only a square patch (area ca. 2 cm^2^) on the dorsal aspect of the rib, in the immediate vicinity of the wound. Projectile fragments extend into the cancellous interior of the rib, with trailing portions of fragments protruding outside of the cortical bone. (**A**) Oblique view in the anterolateral direction, looking upward from just below the level of the cortical bone on the dorsal aspect of the rib. Projectile trajectory from the top right. (**B**) View looking posteriorly, looking upward from just below the level of the cortical bone on the dorsal aspect of the rib. Projectile trajectory from the top left. (**C**) Posterodorsal aspect of the wound area, looking from above. Proximal direction on the rib toward the bottom of the image. (**D**) Dorsolateral aspect of the wound area, looking approximately along the trajectory of the projectile intrusion.

**Fig. 4.**
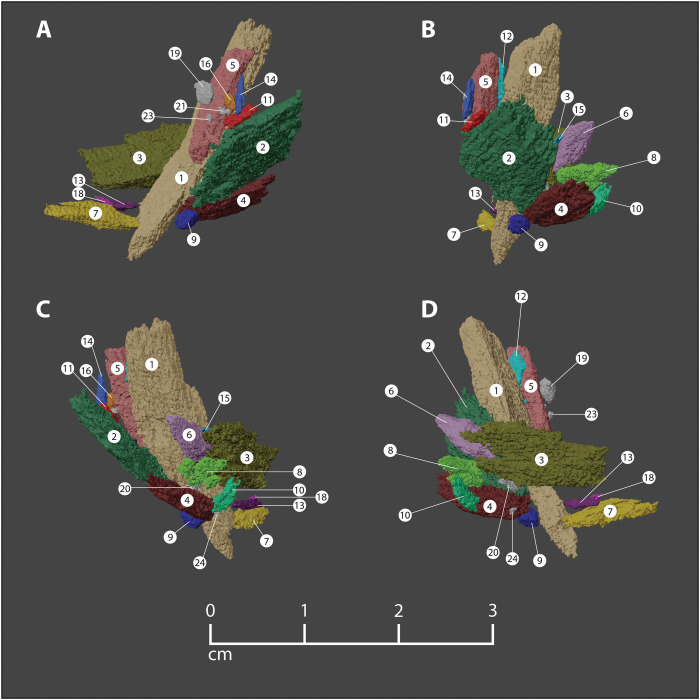
Original orientation and position of point fragments embedded in the Manis rib. All colored pieces were used in the final reconstruction (Fig. 5), while gray pieces were not refitted and do not appear in Fig. 5. (**A** to **D**) The fragments as they are rotated clockwise. Colors and numbers of each fragment correlate with the data presented in table S1.

**Fig. 5.**
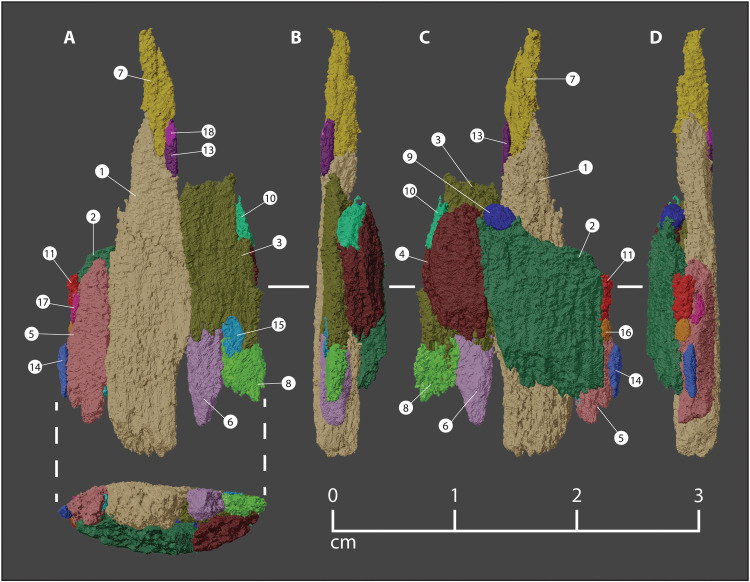
Manis osseous projectile point reconstruction. (**A**) Ventral face and plano-convex cross section. (**B**) Left lateral margin. (**C**) Dorsal face. (**D**) Right lateral margin. Fragment colors and numbers correspond to the data presented in table S1.

**Fig. 6.**
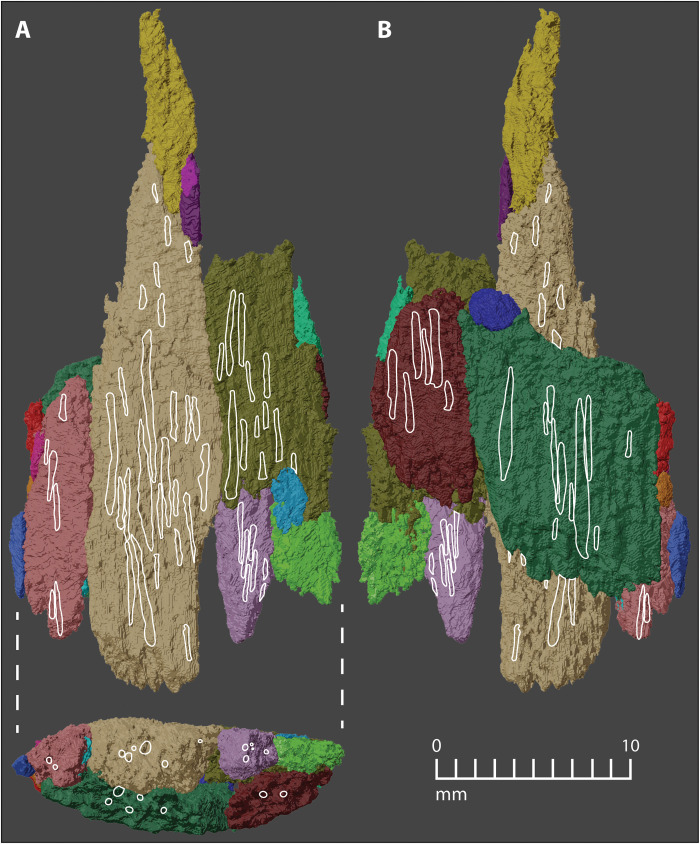
Osseous projectile reconstruction with outlines of internal Haversian canals superimposed. Haversian canals display consistent directions throughout the reconstruction. (**A**) Ventral face and plano-convex cross section. (**B**) Dorsal face. Projectile fragment colors as in Figs. 3 to 5 and table S1. Detailed Haversian canal reconstructions can be seen in fig. S1.

For the current study, we obtained 2067 new high-resolution micro-CT slices (spaced 59 μm apart) of the rib segment containing the embedded fragments (Fig. 2). The CT images revealed that the only foreign material within the cancellous bone of the rib showed x-ray attenuation identical to cortical bone, with thicknesses greater than the cortical bone of the host rib. No material with grain size or texture attributable to sand, silt, or clay is present in the CT images. Using imaging software, we identified 24 individual foreign bone fragments in the rib and generated accurate three-dimensional (3D) models of these pieces (Figs. 3 and 4 and table S1). All fragments were 3D-printed, and 18 of them, representing 99% of the total volume of the foreign bone fragments, were manually refit to reconstruct the morphology of the embedded bone object before it splintered. The six bone fragments that were not refitted were too small or amorphous to permit an accurate match to the reconstructed piece and together comprise only 1% of the total volume of intrusive cortical bone. After the fragments were manually refit, the digital renderings of each bone fragment were digitally refit (Fig. 5). Our digital reconstruction replicated the physical reconstruction. To check the accuracy of our reconstruction, we documented the positions and orientations of internal cylindrical channels that we recognized as Haversian canals within the bone fragments. These occur in cortical bone, are oriented in the same direction, and run parallel to the long axis of the bone. The Haversian canals in the six largest fragments are all parallel to one another, corroborating the reconstruction as accurate (Fig. 6 and fig. S1). See Materials and Methods for more details.

## RESULTS

The reconstructed bone object is 34.5 mm long, 16.9 mm wide, and 5.8 mm thick (Fig. 5). The proximal half of the recovered object is largely complete, whereas the distal half is composed of a central shaft with lateral margins missing. Two burin-like fractures extend 13.5 and 19 mm from the distal end of the object. The proximal half of the object has a plano-convex cross section. The ventral face of the object (Fig. 5A) is relatively flat, whereas the dorsal face (Fig. 5C) is convex. The juncture of the flat and convex faces along the left lateral margin (Fig. 5B) is positioned closer to the flat (ventral) face, while the right lateral margin (Fig. 5D) is more equidistant from the dorsal and ventral surfaces. Both the left and right lateral margins form a sharp edge with an acute edge angle. Both margins also converge toward the midline distally.

**Fig. 7.**
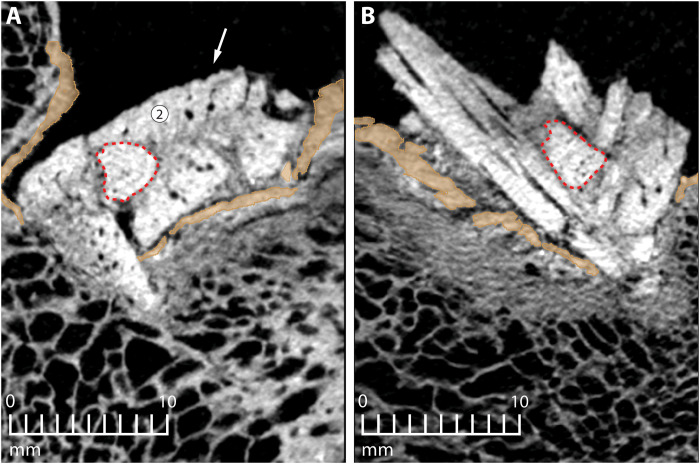
Trabecular bone compaction and bone co-ossification. Displaced cortical bone shown in brown. Red dotted lines outline the cast left behind at the sample location for bone powder used in ancient DNA and protein sequence analysis. (**A**) CT cross section of embedded fragments (white, high-attenuation cortical bone) demonstrating signs of co-ossification (fine, gray texture, indicating lower attenuation, with some void spaces remaining) between discrete fragments. Arrow points to evidence of external striations on fragment 2. Haversian canals appear as small (<1 mm in diameter), roughly circular, black (air-filled) openings in cortical bone. (**B**) CT cross section shows signs of healing between discrete fragments from another angle, along with crushed, co-ossified trabecular bone (fine, gray texture, with only rare voids) beneath the displaced cortical bone.

Examination of the bone fragments protruding from the rib shows that the exterior surfaces of the bone are smooth (Fig. 1 and fig. S2). The graininess evident in the figures is a product of the voxels used in the digital reconstruction (see Materials and Methods). The ventral flat face of the reconstructed fragment (Fig. 5A) is smooth and featureless, but the dorsal convex face (Fig. 5C) has striations that run lengthwise across some portions of the surface. These striations are most pronounced on the right side of fragment 2, but they also exist on fragment 4 (Fig. 5C). They are not a consequence of voxel size or shape because the grooves are deeper than the scale of the gritty texture, and they are visible on CT images (Fig. 7A). On average, the striations are spaced approximately 0.82 mm apart and penetrate 0.16 mm into the surface of the fragment—about 4.5 times deeper than the mean relief produced by protruding voxel corners (~0.035 mm).

The object is dense cortical bone that is 5.8 mm thick. Haversian canals are present across the entire thickness of the bone from one broad face to the other (Fig. 6A, bottom, and fig. S1). These Haversian canals are predominantly straight, run parallel to one another, and show marked variability in their diameters, their spacing (distances between canals, measured perpendicular to their long axes, best seen in canals exposed in transverse cross section; Figs. 6A and 7A and fig. S1), and their lengths (best seen when viewed perpendicular to their long axes; Fig. 6A and fig. S1). This dimensional variability is an indirect reflection of active remodeling of osteons, which truncates old Haversian canals and initiates new ones. This suite of traits is characteristic of cortical bone from a long bone diaphysis of an adult animal undergoing active bone remodeling (*21*, *22*). The 2011 genetic and protein sequence data show that the embedded object is mastodon bone (*16*). The sample used for the molecular analysis in 2011 was powder collected by drilling into the center of the cluster of bone fragments. During our current study, we identified the drill hole. The hole is 4 mm in diameter and extends into the center of the splintered bone object, sampling foreign bone fragments, rib trabecular bone, and bone co-ossification occupying some of the interstices (Fig. 7). This shows that the sample used for the DNA analysis included material from the foreign bone and the host rib. The molecular signature of mastodon, without other animal DNA, shows that both the host bone and the intrusive bone are mastodon.

**Fig. 8.**
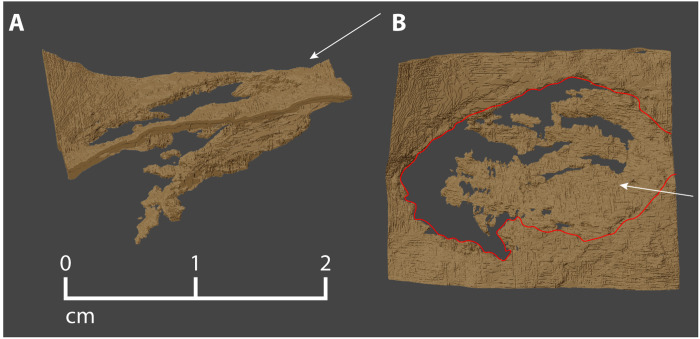
Manis entry wound. (**A**) The wound is the roughly elliptical opening in the solid, dorsal surface of the cortical bone, below which is the inwardly displaced cortical bone that flexed ventrally as the point penetrated into the rib. (**B**) View looking down on the dorsal surface of the rib, with the entry wound outlined in red. The proximodistal axis of the rib is horizontal in this view, with the proximal direction toward the left. White arrows indicate the approximate trajectory of the projectile point.

For this study, we also reconstructed the wound created where the bone object entered the rib. The entry wound is oval in shape and measures 20.2 mm by 9.86 mm (Fig. 8). The wound margins are sharp, with no fracturing or splintering along their edges. The edge of the wound farthest from the rib head merges continuously with an oval region of depressed cortical bone attached to it. This depressed section retains much of its integrity, despite several fractures along its depressed margin (Fig. 8B), and its surface has the same dimensions as the wound opening. Just below this layer of cortical bone is a zone that we interpret as formed predominantly by compacted trabecular bone and reactive tissue that developed as part of the healing response of the Manis mastodon when it was alive. This appears to be a relatively solid, medium-density, hemispherical mass, which appears gray (i.e., has a lower index of x-ray attenuation) compared to the bright white of cortical bone. We interpret the size, shape, and placement of this entire zone as an indication of the existence and extent of a hematoma that formed as an immediate tissue-level response to the trauma of penetration of the rib and disruption of its trabeculae most subject to fracture and displacement (*23*). The thin dark gray zones within this mass appear to represent early stages of co-ossification (discussed further in the Supplementary Materials). Had the Manis mastodon lived longer after its injury, this process would have progressed further, and the “mottled” effect we see in Fig. 7B would have become less pronounced, as overall attenuation increased with progressive ossification. The variation in bone structure and texture revealed here represents a normal response to an injury, requiring at most a few weeks of healing to have attained the degree of stabilization we observe.

## DISCUSSION

There are several key findings derived from this and previous studies that allow us to determine the origin of the bone fragments embedded in the Manis mastodon rib. The fragments became embedded in the rib when the mastodon was alive, as indicated by the visible healing around the site of the wound. The genetic study showed that the bone fragments embedded in the rib are from a mastodon. The CT study shows that the bone fragments and thus the object we reconstructed from them was made of cortical bone. Because the faces of this object are smooth, we considered the possibility that it could potentially be a bone from the mastodon’s own skeleton, but such a bone would have to have the morphology of the reconstructed object. However, there are no mastodon skeletal elements that are the size and morphology of the reconstructed object (*24*–*26*). This tells us that the embedded bone could not be from the Manis mastodon’s own body. It must be from some source external to this animal.

We now gather together the most important traits for interpreting the object that we have reconstructed from the fragments embedded in the Manis rib. Its overall morphology is a central feature—a plano-convex cross section, with both faces converging along both lateral margins to form acute edge angles, and those lateral margins converge distally. Beyond this general shape, the surfaces of the reconstructed bone object are smooth, and the dorsal face has striations that were discussed above. These smooth surfaces and the striations on the dorsal face appear to be the result of intentional shaping of the object by an abrader. Further, the bone object is made of cortical bone that was at least 5.8 mm thick, 16.9 mm wide, and 34.5 mm long. These size parameters, combined with the characteristics of the Haversian canals described above, indicate that the cortical bone was derived from a long bone diaphysis (*22*) of an adult mastodon. On the basis of these key points, we conclude that the bone fragments embedded in the rib of the Manis mastodon are remnants of a human-made projectile point. The three alternative hypotheses proposed to explain the mechanism by which the bone fragments became lodged in the rib are rejected by the data as discussed in the Supplementary Materials.

**Fig. 9.**
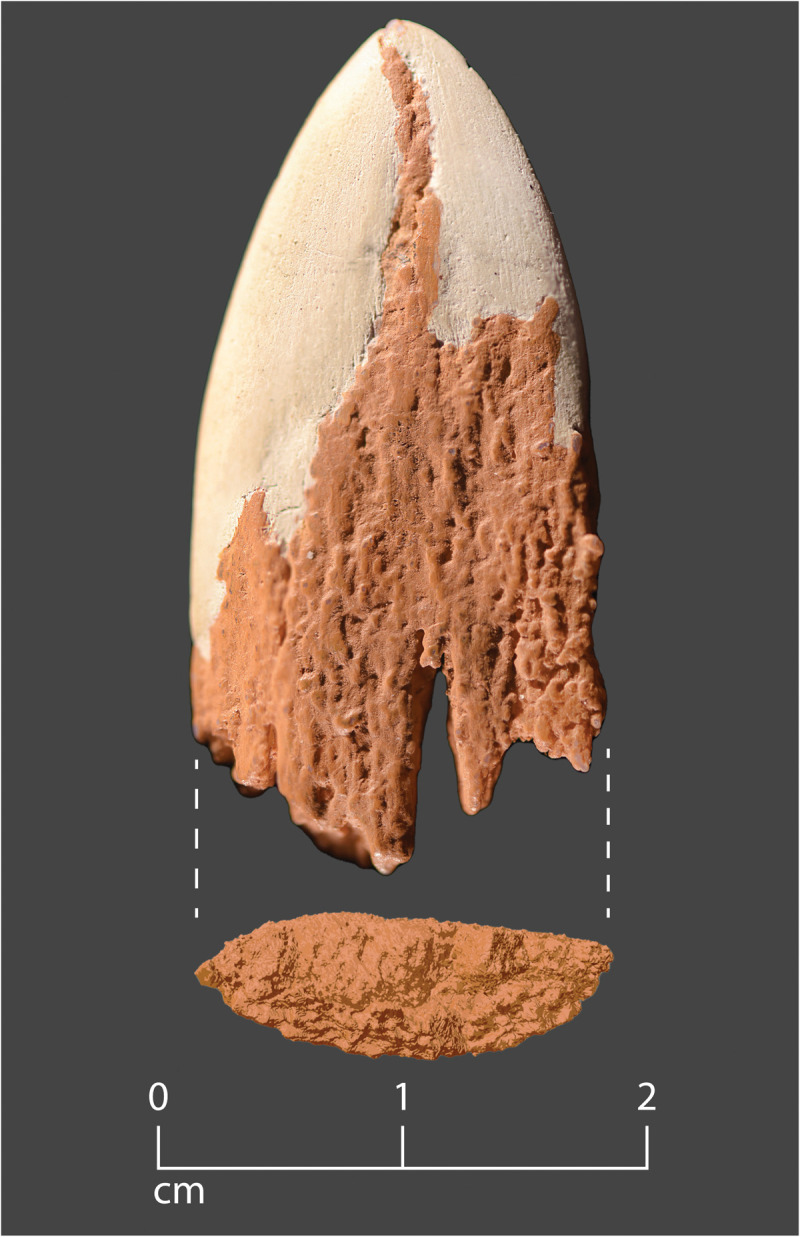
Reconstruction of the distal end of the Manis projectile point. The base of the reconstruction consists of a 3D print of the reconstructed bone fragment that is painted brown. The ventral face is shown (see Fig. 5). The missing portions of the distal end were approximated with white modeling clay. The proximal portion of the projectile point (of unknown length) appears to have broken off on impact. Note the plano-convex cross section of the point.

While it is not possible to know exactly what the entire projectile point looked like, the part we have reconstructed is the tip of the point or close to it, with lateral margins that converged toward its distal extremity. The distal taper and the broad, plano-convex form of the mid-section are reminiscent of the shape of a lithic projectile point (Fig. 9). It is not like the cylindrical osseous points of Clovis and later Paleoindian complexes (*1*).

We envision the osseous point embedded in the rib as the tip of a longer point that was directly attached to a spear or to a foreshaft that socketed into the spear. This spear must have been thrown at the mastodon by a prehistoric hunter with the objective of having the point travel between the ribs, into the body cavity, to puncture a vital organ. Proboscidean lungs extend farther posteriorly than the lungs of most mammals, so this injury is well placed to compromise lung function, if only the projectile had not struck the rib (*27*). Upon impact with the mastodon, the osseous point likely remained intact as it penetrated hide and muscle, but when it hit the rib, the lateral edges of the tip burinated and probably lodged in soft tissue and were thus not preserved in conjunction with the rib. The forward momentum of the spear pushed the thicker, broader, central portion of the distal part of the projectile into the bone, depressing a section of the rib’s cortical bone into its cancellous medullary space, resulting in compaction of the underlying trabecular bone. As the projectile continued into the bone, it encountered resistance from compacted trabecular debris. Fragments of the point then separated along fractures created by the impact and rotated slightly in response to this resistance (Fig. 3). The more proximal portion of the point must have separated from the tip upon impact and was either not preserved or not recovered.

After the projectile point became embedded in the rib, a hematoma formed, and healing began around the bone fragments within the wound. These interpretations (discussed further in the Supplementary Materials), supplemented by information provided by Gustafson, imply that the Manis mastodon was attacked, was wounded, and got away, and the injury began to heal. After that, the Manis mastodon either died of natural causes after the attack and was subsequently scavenged by humans, or it was killed by humans during a second attack and butchered. This corroborates the conclusion of Gustafson *et al.* (*12*) but revises the interpretation of Waters *et al.* (*16*).

The Manis point, made of mastodon bone, shows that people hunted megafauna with osseous weaponry in the Pacific Northwest some 900 years before the emergence of Clovis technology. The use of osseous weaponry like the Manis point and the different stone projectile points of this age found at sites across the rest of North America may signal that the earliest people to enter and explore the Americas brought with them a diversity of technologies and tools that they adapted to the local environments they found and inhabited. The standardized lithic Clovis point form signals the first almost pancontinental weaponry at ~13,050 cal yr B.P.—the meaning of which remains elusive (*28*). After 12,750 cal yr B.P., diversification of projectile point forms again occurs across the continent during the rest of the Younger Dryas.

The reconstructed point from the Manis site is a unique artifact that could be easily overlooked at most paleontological and many archaeological sites. The Manis point was only preserved and brought to our attention because it was embedded in the rib of the Manis mastodon. The Manis projectile point forces us to change our perceptions of what technology we might expect from sites predating 13,000 cal yr B.P.

## MATERIALS AND METHODS

We obtained a new high-resolution micro-CT scan of the rib segment containing the embedded bone fragments using a NorthStar Imaging-NSI X50 CT system located in the Cardiovascular Pathology Laboratory at Texas A&M University. This new scan used a voltage of 130 kV, an amperage of 170 μA, and an integration time of 1.5 frames/s. No filter was used, and the reconstruction was based on 3077 projections. The scan produced 2067 2D images depicting a series of evenly spaced cross sections of the rib, herein referred to as slices, at 0.05901-mm intervals. These images were processed with the open source biomedical imaging software, 3D Slicer. Using the interactive viewport in 3D Slicer, we manually identified individual foreign bone fragments in the rib and generated an accurate 3D rendering of each bone fragment (Fig. 2) through a process called segmentation. During segmentation, once an individual bone fragment was identified on a 2D slice, the area of its cross section was marked using a single layer of 3D voxels, the base unit of graphic information used to characterize the volume and shape of each fragment. This process was repeated for each sequential slice where the fragment appeared. The dimensions of these voxels were set to be equal to the distance between slices (0.05901 mm), so they stack without gaps to form 3D volumetric models of each individual bone fragment.

Our approach ultimately allowed us to document the size, shape, and orientation of 24 individual foreign bone fragments, along with their 3D relationships to each other (Figs. 3 and 4 and table S1). To achieve the most accurate segmentation possible under our scanning parameters, >500 hours were spent manually segmenting the fragments on each of the CT slices, as automated segmentation techniques struggled to distinguish between the closely associated host bone and foreign bone fragments.

After the bone fragments were segmented in 3D Slicer, the 3D models of the 24 pieces were printed at a 6:1 scale for refitting. Physical refitting of these pieces was the first step in the process of determining the projectile point’s original morphology. Demonstrating that these fragments refit using physical models was a critical step because purely digital attempts to refit 3D models carry the risk of overlapping surfaces in ways that would be impossible in real 3D space. The physically refitted model then served as a template for future digital refits. Each fragment has complex morphology with curved faces, variable thicknesses, and irregular edges. Of the 24 foreign bone fragments embedded in the rib (Figs. 3 to 5 and table S1), 18 fragments (99% of the total volume of all intrusive fragments) were unequivocally refitted and resulted in a reconstruction of the foreign object before it splintered. We are confident in our refits because (i) refitted bone fragments on both sides of a mended fracture are similar in thickness; (ii) refitted bone fragments fit snugly along curvilinear and irregular fractures; (iii) refitted bone fragments fit snugly along their fractured edges, with one fragment typically having a convex edge that matches the concave edge of the adjoining piece; and (iv) multiple refitted pieces along the same break interlock with one another. Refitted fragments of bone were always adjacent to one another in the configuration observed in the original specimen. Six bone fragments could not be refitted because they were either too small or amorphous to permit an accurate match to the reconstructed piece. The combined volume of the six excluded fragments represents only 1% of the total volume of foreign bone fragments identified.

Once the fragments were physically refitted, the digital models of each bone fragment were exported to a 3D modeling program called Blender so that they could be digitally refitted in a manner that reflects the physical refit. To further test the accuracy of our reconstruction, we then checked the orientation of internal structures within the bone fragments. During the segmentation process, we observed and marked a series of naturally occurring cylindrical channels, called Haversian canals, within each of the six largest fragments (Fig. 6 and fig. S1). Haversian canals are passageways for blood vessels and nerves in compact cortical bone, and they lie at the center of the basic unit of cortical bone, the osteon. Critically, osteons (and their central Haversian canals) consistently run in the same direction, parallel to the long bone diaphysis (*21*, *22*). The positions and orientations of these canals were then traced in our final digital reconstruction and were consistently parallel (Fig. 6). Any deviations from this pattern would have indicated an inconsistency, but in our reconstruction, all canals run parallel to one another, corroborating the reconstruction as accurate.

In addition to segmenting the individual fragments of bone in the rib and refitting them to reconstruct the intrusive object, the site of the entry wound was also segmented. This helped to confirm that there were no pieces of displaced rib cortex included in the reconstruction of the intrusive object. It also defined the extent of the wound and constrained the entry angles (azimuth and plunge, relative to rib anatomy) of the foreign object (Fig. 8).

## Supplementary Materials

### This PDF file includes:

Supplementary Text Figs. S1 to S4 Table S1 URL for three-dimensional digital model References
