# Supplementary material for: Late Pleistocene osseous projectile point from the Manis site, Washington—Mastodon hunting in the Pacific Northwest 13,900 years ago
Source: Sci Adv. 2023 Feb 1;9(5):eade9068. doi: 10.1126/sciadv.ade9068 (PMC9891687; doi:10.1126/sciadv.ade9068)
Supplement: Supplementary file 1 — Supplementary Text Figs. S1 to S4 Table S1 URL for three-dimensional digital model References [file sciadv.ade9068_sm.pdf]

Science Advances
AAAS

Supplementary Materials for

Late Pleistocene osseous projectile point from the Manis site, Washington—Mastodon hunting in the Pacific Northwest 13,900 years ago

Michael R. Waters *et al.*

Corresponding author: Michael R. Waters, mwaters@tamu.edu

Sci. Adv. **9**, eade9068 (2023)
DOI: 10.1126/sciadv.ade9068

This PDF file includes:

Supplementary Text
Figs. S1 to S4
Table S1
URL for three-dimensional digital model
References

Co-ossification Fabric in the Cancellous Interior of Manis Right Rib 14

In the Results section of our Main Text, we describe the site of the wound in the Manis right rib 14 as including a "hemispherical mass" (Fig. 2B and D) that we interpret as "a hematoma that formed as an immediate tissue-level response to the trauma of penetration of the rib and disruption of its trabeculae". On close inspection, the texture of this hemisphere is not homogeneous. Figure 7B presents a higher magnification view, where we see that it consists of irregular, but roughly parallel, brighter (light gray) elements with an index of X-ray attenuation similar to that of un-compacted trabeculae elsewhere in the rib cross-section. Between the light gray layers are layers that are dark gray in color. Their lower index of X-ray attenuation, other things being equal, implies lower density, and we interpret them as incipient ossification of newly-formed bone, connecting compacted trabeculae. Within this zone of heterogeneous tissue are few voids, which are black in the scan images and air-filled in the specimen as recovered (these would have been occupied by lipid-rich medullary tissue in the living animal). Except for these voids, there are almost no remnant interstices between compacted trabeculae and intervening traces of co-ossification. We take this as an indication of the rapidity of the healing process in this part of the wound, where all fragments are autologous (formed originally by the Manis mastodon and recognized by his immune system as his own tissue).

We see a different response in Figure 2A, where long, slender fragments of the intrusive projectile point penetrate most deeply into the interior of the rib. This is outside the bounds of the hematoma around compacted trabeculae, and no co-ossification reaction was induced by the heterologous, or allogenic (i.e., formed in and by another organism) cortical bone of the projectile point. Yet another response is evident when we move outside the rib's own cortical bone and into the cluster of projectile point fragments. These are again heterologous, but unlike the fragments that penetrated deeply into the rib interior, they would have been in close contact with the rib's periosteum and with injured muscle and connective tissue just outside the rib, which would have been a site of inflammation. Periosteum in this context frequently mediates formation of a bone callus that connects separate fragments, and in Figure 7A and 7B, mineralized tissue appears to have formed where the periosteum and heterologous fragments were juxtaposed. Unlike the mottled texture observed in the rib interior, larger interstices remain in and around the allogenic projectile point fragments, leaving them incompletely conjoined, and yet effectively buttressed and interlocked, explaining why they did not come loose during the drilling that preceded our molecular assay.

This variety of fine-scale textural variation is consistent with the expected inflammatory and immune responses generated in a live animal responding to skeletal trauma involving intrusion of bone derived from a different individual. These observations on immune responses also corroborate our earlier conclusion that the object we reconstructed from the bone fragments embedded in the Manis rib did not have a morphology that matched any known whole bone of a mastodon. At the same time, these observations are profoundly incompatible with a hypothesis (discussed below) in which a cluster of cortical bone fragments embedded in a rib is viewed as resulting from a postmortem (indeed, a modern-day, machine-induced) event.

Evaluation of Alternative Hypotheses for Bone Fragments Embedded in the Manis Rib

Authors commenting on the Manis rib and its intrusive fragments have come up with a range of explanations that differed from those of Gustafson et al. (*12*), Waters et al. (*16*), and the additional, new interpretations presented in our Main Text. These alternative perspectives now require explicit evaluation.

Alternative Hypothesis 1: Elk Antler Hypothesis

The first alternative interpretation was suggested by G. Haynes (*2*), who proposed that the object embedded in the Manis rib was "... an elk-antler tine that the mastodont unwisely rolled on, or even the tip of an antler that an irritated live elk somehow stuck in the mastodon." However, both of these variations are refuted by the DNA analysis and protein sequence analysis of fragments embedded in the Manis rib, showing that they were from a mastodon (*16*). If elk DNA had been present, it would have been detected. Further, elk antler tines do not have a thin, plano-convex cross-section.

By 2015, Haynes had accepted that the object embedded in the Manis rib was derived from a mastodon. However, Haynes broadened the range of tissues under consideration by describing the embedded object as made of "bone or tusk (determined from DNA analysis)" (*18*). Judging from the context of this statement, he may have wondered whether the DNA and protein sequence results reported by Waters et al. (*16*) would have distinguished between these two options, since both could have yielded the same DNA signal, and thanks to the collagen matrix of both bone and dentin, the same protein composition. In addition, the subsequent paragraphs of Haynes' discussion make it clear that he was comparing the Manis case to other examples of bone and ivory projectile points (citing African, Siberian, and North American examples). Although we appreciate Haynes' willingness to consider the possibility of an ivory projectile point, our current results reject this option. If the object embedded in the Manis rib had been made of ivory, transverse fractures on any of the protruding fragments would be expected to show Schreger patterns (reflecting patterns of variation in undulations of dentinal tubules; *29*), but none do. Instead, the Haversian canals we described within fragments embedded in the Manis rib are characteristic of cortical bone in mammalian long bone diaphyses, allowing us to diagnose this object as made of bone.

Reading Haynes' 2015 argument, (elements of which also show up in 2017; *19*) we understand that his unwillingness to accept the Manis object as a projectile point was based on an assumption that the largest bone fragment embedded in the rib was representative of the entire cross-section of the object, which he described as "rectangular in cross section rather than circular…, with maximum width and thickness … of about 3 × 4 mm" (*18*). Citing multiple examples, he argued that this was too slender and lacked "traces of grinding or smoothing … typical of … piercing implements from other places and times in prehistory" (*18*). If the largest Manis fragment had been all we had found, we might have reached a similar conclusion, but given access to the 18 largest fragments, and the means to refit them, we recognized quickly that we were dealing with a substantially larger object, and one that had a smooth profile, with regular grooves on its dorsal (convex) surface. These grooves, as we have argued above, are best understood as evidence of intentional shaping of the object. Perhaps most important, by showing

that the object embedded in the Manis rib is larger than Haynes realized, and that it shows signs of intentional shaping (that he could not have seen because they involve diminutive details of individual fragments), we have now brought to light two critical aspects of the Manis object that might have made a major difference in his assessment.

Alternative Hypothesis 2: Backhoe Impact Hypothesis

In 2015, Haynes (*18*) opened his description of the object embedded in the Manis rib by referring to "The protruding piece …", as if there was only one element to be seen, despite the fact that other fragments are clearly visible in the two photos (a posterior aspect above, and an anterior aspect below) he includes in his figure 8. These other fragments (which are some of the fragments we refitted) receive no mention in his text, but are briefly acknowledged in the last sentence of his caption to figure 8: "Note … also what may be additional bone material inserted into the rib or pushed out of it, alongside the object" (i.e., the single, putative "point"). In this work, Haynes gives us no further insight into how he thinks bone fragments might have been either forced into or out of the rib. Speaking from the perspective of our study, all of the fragments that we segmented, modeled, and reassembled consisted of dense cortical bone, and all of the largest fragments displayed multiple, parallel Haversian canals. There is no chance whatsoever that such fragments came from the cancellous interior of a rib with a cortical zone as thin as that on the Manis right rib 14. These fragments can only have been derived from an extrinsic source.

In 2016, C. V. Haynes, Jr. & Huckell (*20*) also joined the conversation. Their comments largely followed those of Haynes (*18*), whom they cited. They credit Waters et al. 2011 (*16*) with clarifying the geologic age of Manis and providing "bone protein and DNA analyses [that] show both the bone point jammed into the rib and the rib itself are … mastodon" (*20*). C. V. Haynes, Jr. is at first said to have suggested that the Manis rib and its embedded object "could be the result of the backhoe mashing one piece of bone into another" (*20*). However, Haynes "was informed that the director of the radiology lab … had declared that there was evidence of post-trauma healing", and they acknowledged that this would preclude "the backhoe scenario" (20). It was only the published statement by Waters et al. (*16*) that "There is no evidence of bone growth around the point" that reopened the possibility that the backhoe was responsible for emplacing the still un-identified object in the Manis right rib 14.

We are fully aware of the awkwardness of this reversal of interpretation. However, our current CT data are greatly improved over what we had access to in 2011. We now consider the evidence of healing around the object in the Manis rib to be unequivocal.

In 2017, Haynes' latest information from us was dismissive of healing. At this time he acknowledged only two fragments in the Manis right rib 14: "A fragment of a mastodon rib had a thin pointed splinter of mastodon bone or ivory in it, along with another small flat fragment of bone appearing to be inserted with the splinter" (*19*). Still emphasizing the attenuated proportions of what he saw, and the apparent lack of "shaping or grinding", Haynes does not accept the object as an artifact (*19*). His only proposal regarding the origin of this specimen was expressed in one sentence: "The splintering of a mastodon bone or tusk and the forceful insertion of a fragment into another bone may have been done by mechanical equipment excavating

through enclosing sediments" (*19*). However, the idea that the embedded fragments were forced into the rib by the backhoe on the day of discovery (*19, 20*) is incompatible with core elements of our current presentation.

First, all of the 24 bone fragments we segmented and modeled are firmly connected to each other and to the surrounding bone of the rib. If they had been forced into the rib at the time of excavation, no subsequent process would have consolidated them. Post-excavation desiccation would have resulted in shrinkage of the fragments, loosening their association, if they were not already connected, and no conservation-related consolidation of the specimen was undertaken. Second, neither the broken ends of fragments that protrude from the rib, nor the wound on the rib surface display fresh (i.e., modern) breaks, as would be expected if the fracture and forceful intrusion of the fragments had occurred at the time of excavation. Instead of fresh breaks, the bone fragments protruding from the rib are weathered to the same degree, and exhibit the same pattern of weathering, as the surface of the rib, including its broken distal end (Fig. 1). In particular, dark root stains found on the surface of the bone extend onto exposed portions of the embedded bone. None of this is consistent with a novel association of a rib fragment and a collection of other bone fragments that dates only to the time of excavation. Third, if the bone fragments were driven into the rib by a backhoe, the introduced bone should have been covered in sediment, and clastic grains should be present within the rib; yet the CT slices show no evidence of sediment grains inside the rib. A fourth problem with this explanation is that Haynes (*19*) and Haynes and Huckell (*20*) offer no explanation for how a backhoe could manipulate a bone fragment in such a way as to orient it, and then drive it along its long axis, so as to cause it to penetrate the rib fragment in question. Even if this were explained for one such fragment, the alternative explanations do not address the multiplicity of bone fragments that make up the cluster that we interpret as parts of a projectile point that fractured on impact. The largest number of bone fragments Haynes (*19*) acknowledges is two – well shy of the 24 fragments we report here. Finally, the evidence we report in our Main Text, and in more detail in our supplementary text addressing co-ossification above, demonstrates a physiological response (inflammation and subsequent co-ossification) at the site of the Manis rib injury. The intrusive bone fragments therefore represent an ancient injury, sustained while the mastodon was alive. The detailed patterns of variation in X-ray attenuation that we document in CT slice images and interpret in terms of hematoma formation and incipient co-ossification, cannot be explained away as consequences of backhoe operation many millennia after the death of the animal.

Alternative Hypothesis 3: Musth Behavior Hypothesis

The final suggestion was proposed by Grayson and Meltzer (*3*), who argued that the bone fragments embedded in the 14th right rib of the Manis mastodon could have resulted from an injury sustained when the Manis male fought with another male mastodon during the mating season. They suggested that during such a battle, a piece of bone became dislodged from the Manis mastodon's own skeleton, travelled some distance through muscle and associated tissue, "driven … by the anger of [Manis'] opponent" (*3*), and finally become embedded in the rib fragment that Gustafson recovered. Grayson and Meltzer provide no evidence to support the feasibility of this scenario, but as discussed below, the issue is not whether musth combat has the potential to produce serious injury for one or both combatants.

The phenomenon of “musth” has been observed closely in Asian elephants (*Elephas maximus*) for hundreds of years, throughout the long history of human interaction with captive individuals of this species (*30*). Musth involves a syndrome of behavioral and physiological traits indicative of a heightened state of aggression toward other mature males and urgent pursuit of estrous females. More recently, musth has also been recognized in African savanna elephants (*Loxodonta africana*), where it is characterized by virtually identical manifestations (*31*). Aggressive interactions between musth males in extant elephants do not always escalate to full-blown battles, but when they do, fatal wounds are sometimes inflicted (*32*). Moreover, fossil evidence of musth-like behavior (bone breakage patterns and characteristic styles of tusk damage) has been recognized in late Pleistocene individuals of *M. americanum*. Mastodons interpreted as having engaged in musth battles, and that died from injuries sustained in musth battles, include three relatively complete and well-studied specimens, the Hyde Park, Buesching, and Cohoes mastodons (*33, 34*).

As a first step toward evaluating whether the Manis mastodon is likely to have engaged in musth-like behavior in the years shortly before its death, we remind readers that Gustafson and co-authors (*12*), and all subsequent commentators on this specimen have interpreted it as an adult male. The published information that best supports this interpretation is the description of the specimen as “a very large individual with massive, curving tusks over 2 m in length” (*12*). Near the start of their article, Gustafson et al. clarify that there were two tusks, both broken, and that “the largest segment recovered intact was nearly 2 m in length.” As shown in a graph (text-figure 13B in *31*) that superimposes profile plots of tusk circumferences (at 10-cm intervals) vs. length along the outside curve, for both male and female mastodons of a wide range of sizes, the reported segment length (and implied tusk circumference) for Manis is too great for this specimen to be interpreted as a female. American mastodons display strong sexual dimorphism (*35*), so the Manis mastodon was clearly a male.

A second factor relevant to assessing the likelihood of musth behavior is the Manis mastodon’s age at death. Gustafson and co-authors (*12*) report consulting with Saunders (*36*), who was the first to apply Laws’ Age Groups (for African savanna elephants, *Loxodonta africana*) to estimating biological age at death for *M. americanum*. Since only the second lower molars of Manis were available (access to one or both third lower molars would have permitted greater precision), Saunders quoted a relatively broad assignment of Laws’ Age Groups: XXII through XXIV. Converting this to years (as manifested in the lower dentition of *L. africana*, and gravitating toward the older end of his range of uncertainty), Saunders estimated an age of 45 yr, with the obvious qualification that *M. americanum* might have shown either more or less advanced stages of wear in its dentition than are observed at a given age in *L. africana*. As it happens, Saunders’ placement of Manis in Laws’ Age Group(s) is not much different from assignments that have been published for the three adult males already interpreted as having died in musth battles (Cohoes, XX; Buesching, XXI; and Hyde Park, XXII)(*33*). For two of these, more secure estimates of age at death based on counting annual increments in the longer of two tusks, and on estimating the number of years broken or abraded from the tip of each (based on comparison with a tusk of a much younger male, who had suffered less tip loss) yield age estimates of 36 yr for Hyde Park (*33*) and 34 yr for Buesching, (*37*). Accepting that Manis might have been older at death than the Hyde Park or Buesching mastodons, we are inclined to adjust Manis’ age at death from Saunders’ estimate of 45 yr to a value closer to 38 yr. Moreover, the

tusks of both the Hyde Park and Buesching mastodons show a series of externally visible features (with histological correlates referred to as "cementum defects"; *33*) reflecting damage characteristic of non-lethal musth battles. This record of tusk damage suggests that these animals engaged in musth battles annually, always in the same season (late spring to early summer), for at least a decade prior to death (i.e., from their early 20s onward). Without assuming that any two lives would recapitulate identical schedules, it is indeed likely that Manis experienced musth battles prior to his death.

Having thus established the plausibility of one or more musth encounters prior to Manis' death, we recall that Grayson and Meltzer (*3*) raised the prospect of testing their hypothesis by scanning "the Manis skeleton … to determine if there [was] a missing piece that might match the object in the rib". However, their next sentence reads, "Since the Manis mastodon has never been described in detail, there is currently no way of knowing whether or not this might be the case." Our familiarity with mastodon osteology leads us to estimate the odds of such a discovery as small, but instead of focusing on our limited access to Manis, we consider it no less interesting to examine other skeletons of adult male mastodons known to have engaged in musth battles.

The Hyde Park mastodon (Paleontological Research Institution, PRI 49820) is the most thoroughly studied of these specimens (*33*). It was excavated in eastern New York State by a large and knowledgeable crew with protracted and unrestricted access to the site (*24, 38,39*). The fraction of the Hyde Park skeleton recovered was relatively high, with 214 bones (of about 256 expected) retrieved (*38*), most of them documented in situ (*33*, text-figure 2). One of us (DCF) initiated an institutional loan of all Hyde Park skeletal material, studied and photographed this at length, and supervised molding and casting of every element before returning it to PRI (except for some tusk material still under study).

Most notable among a series of injuries sustained near the time of death is a deep puncture wound in the right temporal fossa of the skull (near the jaw joint) that probably contributed to death and that was apparently caused by an opponent's tusk tip (*33*, text-figure 27E). In addition, the tusks of the Hyde Park mastodon show evidence of damage incurred while either delivering blows to an opponent or parrying blows from an opponent's tusks (*33*, text-figure 28A). The most severe perimortem postcranial injury was caused when an opponent's tusk tip was thrust between two consecutive thoracic vertebrae (T8 and T9), penetrating deeply enough to disrupt the spinal cord, probably causing loss of hind limb locomotor and support function. However, neither of these injuries broke loose any fragment of bone with potential for displacement to permit replication of the intrusive injury to right rib 14 of the Manis mastodon.

The Hyde Park skeleton also shows evidence of multiple, forceful blows to the torso, probably by tusks. Some of these impacts transferred enough energy to cause incipient dislocation of adjacent thoracic vertebrae. Perimortem instances of this style of damage were marked by acute crushing damage at the margins of zygopophyses (where thoracic vertebrae have synovial articulations). Other examples of the same style of damage showed various stages of healing. In all of these cases, the damage to bone is always located deep within the structure of the vertebral column, leaving no route by which fragments of bone could be released from, or projected through, multiple layers of connective tissue and muscle in a way that would allow them to be displaced to another part of the body, even to ribs that articulated with the same

vertebrae. Add to this the fact that the trajectory of entry of the bone projectile that impacted and embedded itself in right rib 14 of the Manis mastodon came from a position dorsal and lateral to the head of the rib, plunging in a ventromedial direction approximately along an azimuth parallel to the long axis of the rib. This is opposite to the direction from vertebral zygopophyses toward the site of impact on right rib 14 of Manis. There is no way in which bone fragments generated by incipient disarticulation of thoracic vertebrae could follow such a trajectory.

The Hyde Park skeleton also preserves rib fractures, some of which had occurred early enough to have totally healed by the time of death, and others that were completely unhealed and no doubt formed close to the time of death. A case of healed fractures consisted of an aligned series of three breaks on left ribs 14-16. These were clearly made, essentially simultaneously, by the outer curve of a tusk oriented obliquely relative to the long axes of these ribs, such that the three fracture sites were progressively offset, proximally or distally, from one rib to the next. No loose bone fragments were produced by these fractures, leaving complementary patterns of undamaged bone on opposite sides of each healed fracture.

An example of an unhealed broken rib was a fracture on right rib 10, about a quarter of the rib's length from its proximal end. This injury shows a distinctive configuration known in medical and forensic literature as a "butterfly fracture" (*40*). Butterfly fractures form under conditions of "three-point bending" (*40, 41*), where the ends of a bone are stabilized against an applied force vector acting roughly perpendicular to the bone. The Hyde Park mastodon's right rib 10 was stabilized proximally (in life) by anatomical connection at the vertebral column, and distally, by compression against the viscera and by tensile properties of tissues connecting the ventral ends of ribs. The rib was probably broken by impact from the ventral aspect of a competitor's tusk, crashing down forcefully in a ventromedial direction. No hint of damage occurs on Hyde Park's right rib 9 or right rib 11, suggesting that the impactor was approximately aligned with all three ribs, releasing most of its energy directly onto the opposed curvature of the dorsolateral aspect of the Hyde Park mastodon's right rib 10. This would have focused the energy of impact on a narrow target, only centimeters across, tending to bend the rib in a direction opposite its natural curvature. This localized straightening, or reverse bending, subjected the visceral surface of the rib to tension (along its axis) and the lateral surface of the same rib to compression. Because bone is stronger under compression than under tension, the rib failed in response to supercritical tensile stress, initiating a transverse fracture that opened on the visceral surface (opposite the applied force) and propagated dorsolaterally, toward the applied ventromedial force vector (figs. S3, S4). When this fracture approached the neutral axis of the rib, where the transition from tensile stress to compressive stress had been located (when bending began), it bifurcated, with one newly initiated fracture plane turning proximally and an opposed fracture plane turning distally, leaving a signature Y-shaped fracture trace on both anterior and posterior aspects of the rib. Both of these fractures propagated outwardly, but in opposite directions along the axis of the rib. The proximal fracture ended as a "greenstick" fracture opening onto the lateral surface of the rib, and the distal fracture died out as it entered a zone of complexly woven bone fabric within the dorsolateral cortex of the rib. Once again, no loose bone fragment was produced by this fracture, and no part of the bone was subject to any degree of translocation, whether through adjacent tissue or along a ballistic trajectory. Even the proximal and distal segments of this rib (separated by the tensile fracture and its dorsal continuation into a greenstick termination) would have remained juxtaposed by gravitational

forces pulling the proximal mass downward against surrounding connective tissue. The only potential opportunity for dislocation of a bone fragment would have arisen if the distal fracture had run to completion on the lateral surface of the rib, which would have broken the butterfly fragment from the rest of the rib. Even in this case, however, the periosteal membrane covering the outer surface of that fragment (not to mention other connective tissue nearby) would have kept all pieces closely associated. Thus, even under circumstances that might seem ideal for producing a loose fragment, the butterfly fragment would only have been subjected to viscerally (medially) directed compressive force from the tusk impact and bending moment. It would never have experienced any proximally directed traction force that could have displaced it toward the proximal end of the rib, where we discovered bone fragments embedded in the dorsolateral aspect of right rib 14 of the Manis mastodon. Further, such a butterfly fragment would not have had a plano-convex cross section like that of our Manis point reconstruction.

The Hyde Park mastodon and other victims of musth battles (e.g., the Buesching and Cohoes mastodons; *34*) support the impression that, with the exception of stereotypical damage to crania, tusks, and some ribs, musth combat is not responsible for extensive bone breakage. The most compelling support for Grayson and Meltzer's hypothesis would be if a bone like the Manis rib, with its embedded object, were to show up at a site preserving remains of another mastodon. Another mastodon with an inferred history of musth combat might seem most relevant, but in fact, any mastodon skeletal element with an embedded fragment of bone would be worthy of study for evidence of its origin. However, nothing like the Manis rib appeared at the Hyde Park site, or at Buesching or Cohoes, all of which appear to represent musth deaths. Broadening the search to mastodons, both male and female, that died of causes unrelated to musth behavior, the answer remains the same. Even bringing our focus to extant elephants, we are aware of no injury comparable to that seen on the Manis rib.

Setting aside our effort to find a bone fragment embedded in another bone of a mastodon, we might instead ask whether indeterminate bone fragments have been reported, with the approximate size and shape of the embedded object at the Manis site? Thus far, we have nothing matching this search image from the Hyde Park mastodon site, or Buesching, Cohoes, or any of the tens of other sites we have studied. Is there instead some whole bone of an appropriate size and shape? Only the left or right stylohyoideum (osteology of the hyoid apparatus is described in *26*, although the interpretation of left and right stylohyoidea is reversed in this reference; *33*) bears a superficial resemblance to the object we have reconstructed, but even one of these (from an adult male; stylohyoidea show marked sexual dimorphism, and those of adult males are more robust; *33*) would require shaping and reduction of mass to approximate the configuration of the hypothesized complete osseous projectile point. The mismatch in mass would actually reverse its polarity if we proposed an association of a female stylohyoideum with an adult male victim (Manis) because a female stylohyoideum is not broad enough to match the breadth of the object we reconstructed. To be sure, we would not expect an adult male and a female mastodon to be fighting, but even if they did, how would one combatant extract a stylohyoideum from the other, retrieving it from the deep mass of tongue musculature in which it occurs, detaching it from the other hyoid elements to which it is connected by ligaments, and then accelerate it toward the right flank (where right rib 14 is located) of its opponent (Manis). Granting that the Hyde Park mastodon is a different case, both stylohyoidea of that animal were recovered, and neither showed any sign of perimortem or postmortem damage.

Combing proboscidean literature for examples of autologous bone fragments embedded in another bone of the same individual was less productive than we had hoped it might be. However, we decided to go further, exploring literature on the effects of high-velocity impacts on the human body during wartime. There is a great deal of information on bone injuries resulting from violent explosions (e.g., of landmines and artillery shells) and impacts (e.g., from cannon balls and bullets), harming soldiers and civilians alike, from the time of the American Civil War to the War in Afghanistan (e.g., *42-44*). However, we still found no examples of what we had come to call "bone-in-bone injuries," such as the one on the Manis mastodon rib.

At what seemed like our "eleventh hour", in an article describing a high-speed vehicular collision, we finally encountered a case that seemed to exemplify Grayson and Meltzer's mechanism. In their introduction, the authors (*45*) acknowledged what we had come to accept as common understanding: "Fractures caused by projectiles of organic tissue are almost unheard of and the few cases present in the literature describe fractures associated with projectiles of *heterologous* [our emphasis] tissue." Instead, the victim of this crash appeared at first to have "sustained an iliac wing fracture caused by a projected bone fragment [that] originated on [a] synchronous femoral fracture." The anatomical context and details of the crash and consequent injures are complex. Under the influence of extreme compressive forces associated with impact, the victim's broken femoral shaft stabbed through one side of his pelvis, after which a fragment of the fractured femoral shaft was left in the fractured pelvis when the victim's limbs were straightened during transport to the hospital. In other words, the autologous bone (femoral) fragment was never a "projectile" in the sense of having been thrown on a ballistic trajectory before reaching its point of intrusion into the pelvis. Rather, the femoral shaft effectively behaved like a spear that was *thrust* into the pelvis, leaving a loose fragment from its tip embedded there when the rest of the femur was withdrawn.

Lest this case seem too bizarre to be relevant to the current debate, we propose that it forces readers to consider seriously what Grayson and Meltzer's suggestion (*3*) really requires in terms of tissue properties, behavior, and associated physics. Most fractures produced during a proboscidean musth battle are likely to result from a tusk impacting some part of an opponent's body. In the special case of tusk-on-tusk impact, a fragment of tusk could easily form almost instantaneously and be released with considerable kinetic energy. However, such an "ivory flake" is likely to separate along a broad fracture front and is unlikely to have the kinetic energy to penetrate soft tissue along a broad contact margin. Penetrating soft tissue deeply enough to hit bone, break through cortical bone, and intrude deeply into a cancellous medullary tract is even more improbable – but again, this is an ivory flake, not what was embedded in the Manis rib.

When a vigorously swung tusk impacts part of an opponent's body that is softer than a tusk, what happens will depend largely on the angle of impact. At a low angle (a "glancing blow") deceleration of the tusk might be modest, allowing it to propel any loose fragment along a tangential trajectory, but what loose fragment will be available? At a higher angle of impact, more of the energy of impact will be transferred to the opponent's body, and more damage may occur. However, bone fragments produced by this blow will probably be compressed inward toward the core of this part of the body, rather than experiencing traction forces that would tend to detach them from surrounding tissue. Most important, the external surface of mammalian bone

(except for joint surfaces) is covered by tough "periosteal membrane" or "periosteum". This does not simply envelope the bone; rather, bone and periosteum are deeply connected by Sharpey's fibers that have both great tensile strength and toughness. Moreover, muscles, ligaments, and other connective tissues are firmly attached to the external surface of the periosteum, with the result that fragments of bone resulting from impact are almost never "loose" at all. To be displaced, forces acting on them would have to be great enough to rupture all of their connections. Further, if such fragments were displaced, the idea that they might, on impact, retain the directional stability to become embedded in another bone of the same animal is, to say the least, improbable. And if that conundrum is not challenging enough, consider the probability that, having broken loose from some part of a mastodon's skeleton, the hypothetical fragment would still be carrying enough energy to shatter on impact with its target bone. In contrast, if, as we propose, the object embedded in right rib 14 of the Manis mastodon is a bone projectile point, originally attached to a spear, the directional stability of the point would tend to be maintained by its connection to the spear … until the final instant when those two components separated by fracture, on impact.

Conclusion

In summary, we have found *no* examples of bone-in-bone injuries caused by natural processes in extant or extinct proboscideans. The only example of a bone-in-bone injury we have found is the Manis mastodon rib itself. DNA and protein sequence analyses (*16*) rule out identifying the embedded object as an elk antler tine (*2*). The morphology of the reconstructed, embedded object is inconsistent with interpreting it as either a fresh fragment of bone produced on the day of the Manis excavation (*18, 19, 20*), or a fragment of bone derived from the mastodon's skeleton during musth combat, when he was alive (*3*). CT evidence of a vital response to the trauma of the object's penetration into the rib rules out the backhoe scenario, but interestingly, is consistent with the musth combat scenario, since in this model, the animal is alive at the time of injury. However, our assessment of the anatomical context of bone fragments produced during musth combat virtually precludes the release of such fragments with enough energy and directional stability for them to become embedded in a rib. Instead, the morphology of the reconstructed, embedded object, the CT evidence of a vital response, and the energy and directional stability required for high-velocity impact and penetration of a fresh rib, are all consistent with expectations for a projectile point mounted on even a light-duty spear. We conclude that this interpretation is best supported by the data.

SUPPLEMENTAL FIGURES

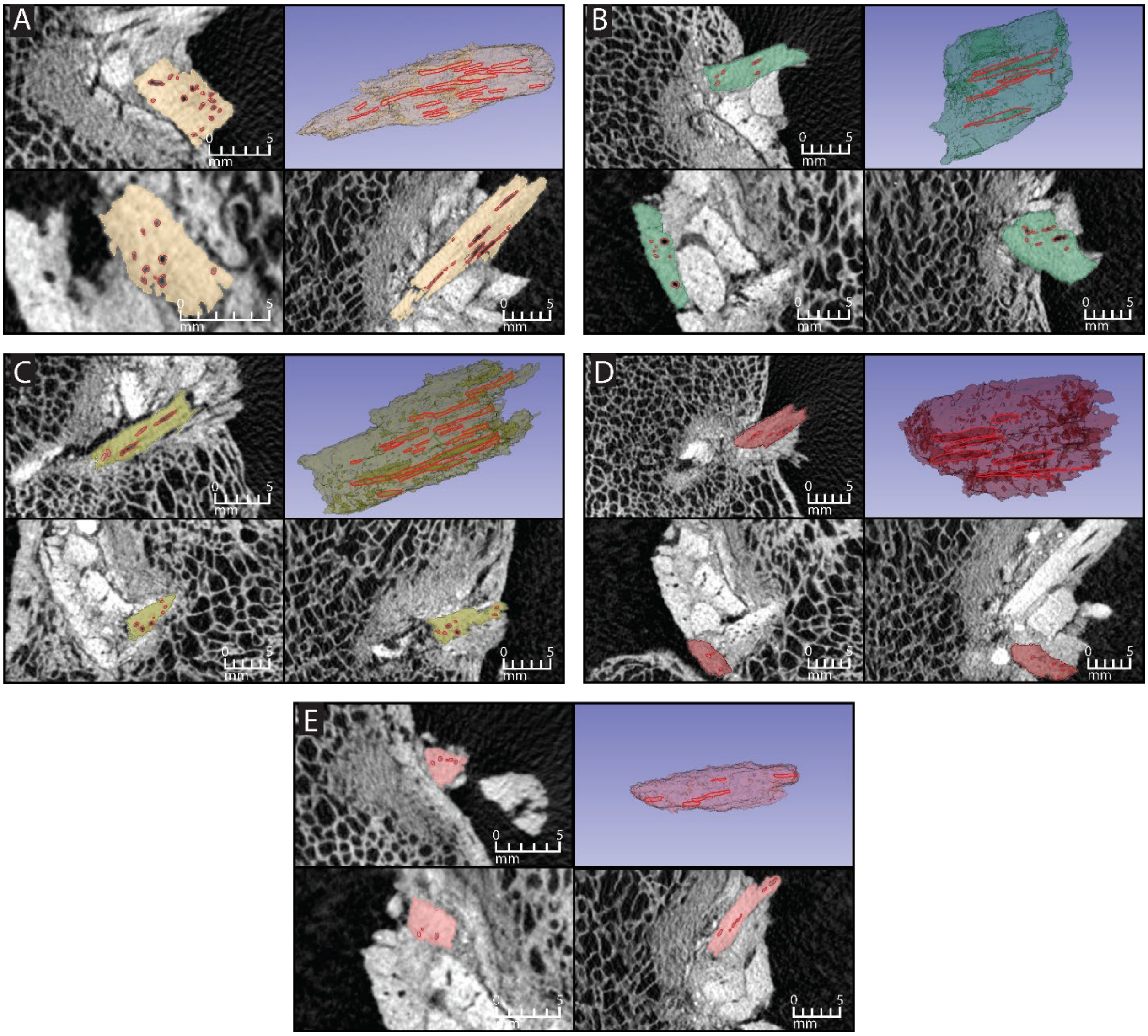

Fig. S1. Outlines of Haversian canals (red) present within various point fragments. A-E depict volumetric renderings of a particular fragment with reduced opacity to reveal internal structure in the upper right-hand corner, along with micro-CT slices of that fragment from three angles: axial (lower right-hand), horizontal (lower left-hand), and transverse (upper left-hand). A. Fragment 1, B. Fragment 2, C. Fragment 3, D. Fragment 4, E. Fragment 5. Colors of fragments depicted in A-E may not match other figures due to adjustments in opacity. Volumetric renderings (top right-hand corners) of A-E not to scale.

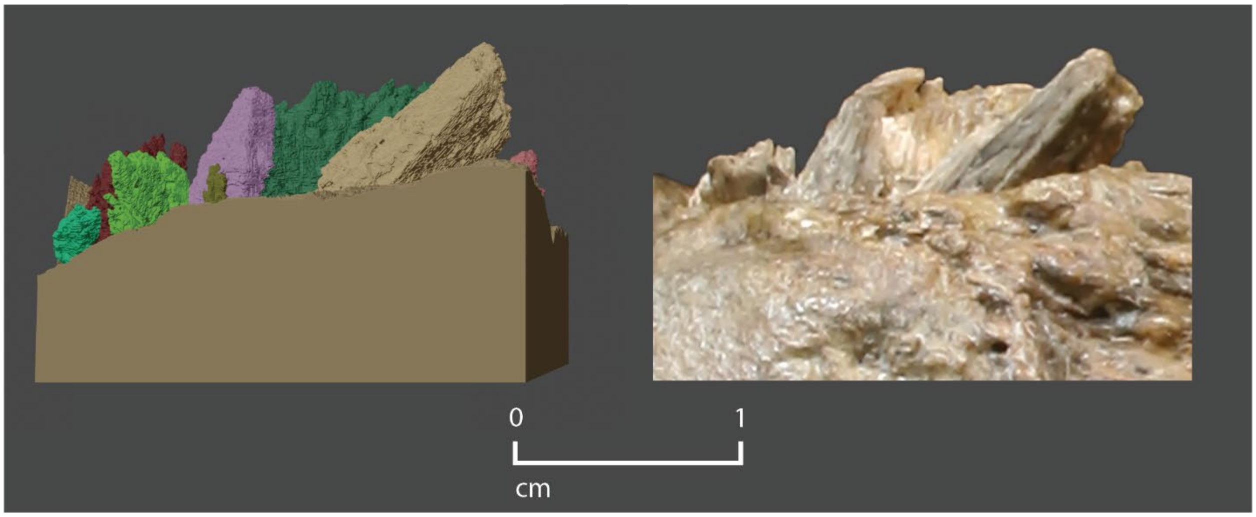

Fig. S2. Three-dimensional model of projectile point fragments compared to photograph. Image shows the three-dimensional model (left) and a photograph of the actual specimen (right) at the same scale from the same perspective to illustrate the degree of graininess produced by the modeling process.

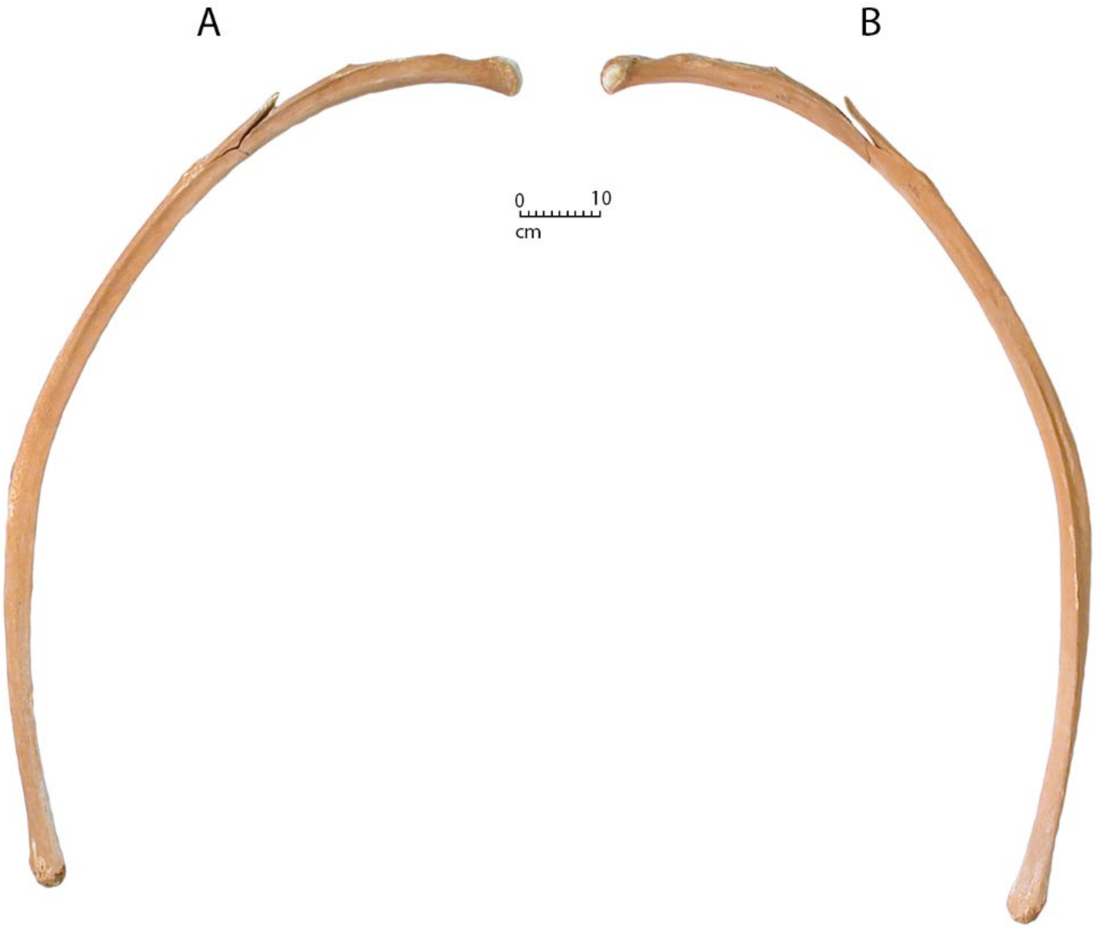

Fig. S3. Right Rib 10 of the Hyde Park mastodon (PRI 49820). Fracture in proximal half of rib is an incomplete butterfly fracture, initiated as a tensile failure on medial aspect of rib, produced by impact bending rib inward from outside the body. A. Anterior aspect of rib, viewed normal to main plane of curvature. B. Posterior aspect of rib, viewed normal to main plane of curvature. Scale for both components between rib images.

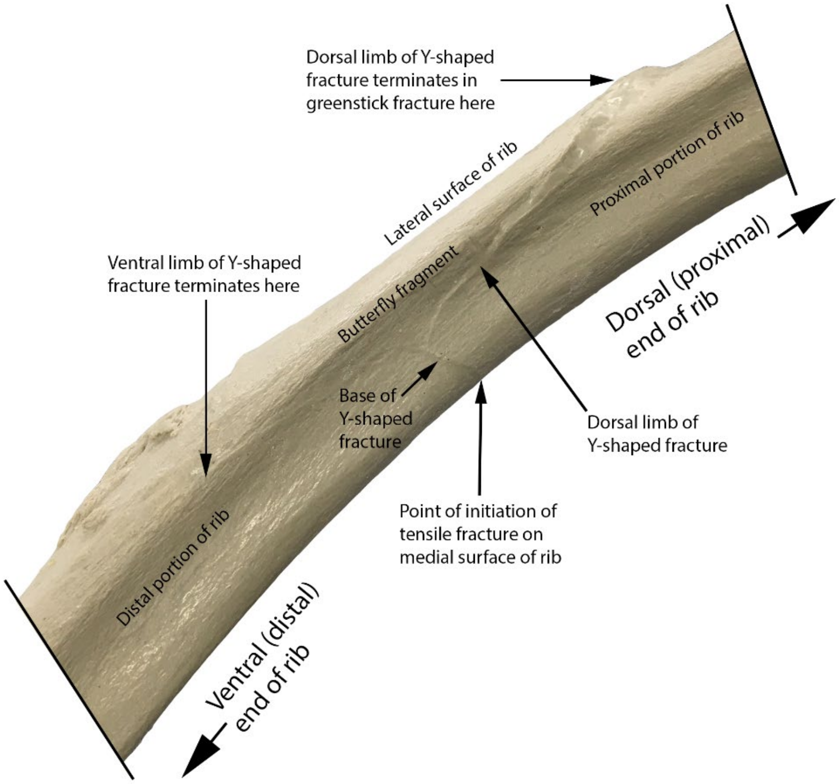

Fig. S4. Fiberglass and polyester resin cast of reassembled Right Rib 10 of the Hyde Park mastodon (PRI 49820). Image shows anterior aspect of part of rib near butterfly fracture. Orientation of this truncated portion of rib matches view in Fig. S3A (but at larger scale; consult Fig. S3A for scale). Fracture features discussed in text.

Table S1. Dimensions of bone projectile point fragments color-coded to match Figures 2-6.

Segment Number	Color	Length (mm)	Width (mm)	Thickness (mm)	Volume (mm^3)	% of Total Volume
1		28.70	6.73	3.39	299.06530	37.29
2		14.10	11.30	2.14	178.73500	22.23
3		17.20	5.67	2.46	101.15400	12.61
4		11.10	5.69	2.09	62.66640	7.81
5		12.60	3.57	1.75	59.52770	7.42
6		8.30	3.07	2.16	29.46430	3.67
7		10.50	2.22	1.86	25.11070	3.13
8		6.43	3.72	1.29	14.14804	1.76
9		2.43	1.94	1.90	5.23116	0.65
10		4.56	1.81	1.40	4.67245	0.58
11		3.90	1.60	0.72	3.82813	0.48
12		6.39	2.29	0.53	2.64558	0.33
13		4.74	1.00	1.00	2.20462	0.27
14		4.32	1.14	0.70	2.08174	0.26
15		3.23	1.55	0.63	1.55200	0.19
16		1.99	0.97	0.80	1.11022	0.14
17		2.50	0.71	0.42	0.56836	0.07
18		2.06	0.86	0.29	0.24288	0.03
19		3.43	1.79	1.59	5.14917	0.64
20		2.89	1.34	0.79	1.97324	0.25
21		1.38	0.66	0.45	0.28192	0.04
22		2.20	0.65	0.35	0.24535	0.03
23		0.93	0.73	0.43	0.17487	0.02
24		0.99	0.75	0.35	0.15925	0.02

URL for Three-dimensional digital model of region near butterfly fracture on Right Rib 10 of the Hyde Park mastodon (PRI 49820): Intersection of the fracture and the outer surface of the rib is traced by a single dark line where fracture surfaces can be easily approximated, and by a double dark line where stress release following fracture resulted in modest deformation of bone, preventing precise approximation of fracture surfaces. Dual articular facets on rib capitulum mark proximal end of rib; opposite end truncated distal to fracture. Responsiveness of model (to 3D manipulation; navigation instructions are displayed when model opens) is optimized when accessed using the browser software Chrome.

https://umorf.ummp.lsa.umich.edu/wp/specimen-data/?Model_ID=1618

REFERENCES AND NOTES

1. B. A. Bradley, M. B. Collins, A. Hemmings, *Clovis Technology* (International Monographs in Prehistory, Archaeological Series 17, 2010).

2. G. Haynes, *The Early Settlement of North America: The Clovis Era* (Cambridge Univ. Press, 2002).

3. D. K. Grayson, D. J. Meltzer, Revisiting Paleoindian exploitation of extinct North American mammals. *J. Archaeol. Sci.* **56**, 177–193 (2015).

4. M. R. Waters, T. W. Stafford Jr., D. L. Carlson, The age of Clovis—13,050 to 12,750 cal yr B.P. *Sci. Adv.* **6**, eaaz0455 (2020).

5. M. R. Waters, J. L. Keene, S. L. Forman, E. R. Prewitt, D. L. Carlson, J. E. Wiederhold, Pre-Clovis projectile points at the Debra L. Friedkin site, Texas—Implications for the late Pleistocene peopling of the Americas. *Sci. Adv.* **4**, eaat4505 (2018).

6. T. J. Williams, M. B. Collins, K. Rodrigues, W. J. Rink, N. Velchoff, A. Keen-Zebert, A. Gilmer, C. D. Frederick, S. J. Ayala, E. R. Prewitt, Evidence of an early projectile point technology in North America at the Gault site, Texas, USA. *Sci. Adv.* **4**, eaar5954 (2018).

7. L. G. Davis, D. B. Madsen, L. Becerra-Valdivia, T. Higham, D. A. Sisson, S. M. Skinner, D. Stueber, A. J. Nyers, A. Keen-Zebert, C. Neudorf, M. Cheyney, M. Izuho, F. Iizuka, S. R. Burns, C. W. Epps, S. C. Willis, I Buvit, Late upper paleolithic occupation at Cooper's Ferry, Idaho, USA, ~16,000 years ago. *Science* **365**, 891–897 (2019).

8. M. R. Waters, Late Pleistocene exploration and settlement of the Americas by modern humans. *Science* **365**, eaat5447 (2019).

9. M. R. Waters, T. W. Stafford Jr., B. Kooyman, L. V. Hills, Late Pleistocene horse and camel hunting at the southern margin of the ice-free corridor: Reassessing the age of Wally's Beach, Canada. *Proc. Natl. Acad. Sci. U.S.A.* **112**, 4263–4267 (2015).

10. J. J. Halligan, M. R. Waters, A. Perrotti, I. J. Owens, J. M. Feinberg, M. D. Bourne, B. Fenerty, B. Winsborough, D. Carlson, D. C. Fisher, T. W. Stafford Jr., J. S. Dunaber, Pre-Clovis occupation

14,550 years ago at the Page-Ladson site, Florida, and the peopling of the Americas. *Sci. Adv.* **2**, e1600375 (2016).

11. D. J. Joyce, Pre-Clovis megafauna butchery sites in the western Great Lakes region, USA, in *Paleoamerican Odyssey*, K. E. Graf, C. V. Ketron, M. R. Waters, Eds. (Texas A&M Univ. Press, 2014), pp. 467–483.

12. C. E. Gustafson, D. Gilbow, R. D. Daugherty, The Manis Mastodon site: Early man on the Olympic Peninsula. *Can. J. Archaeol.* **3**, 157–164 (1979).

13. K. L. Peterson, P. J. Mehringer Jr., C. E. Gustafson, Late glacial vegetation and climate: The Manis mastodon site, Olympic Peninsula, Washington. *Quat. Res.* **20**, 215–231 (1983).

14. D. W. Gilbow, "Inference of human activity from faunal remains," thesis, Washington State University, Pullman, WA (1981).

15. A. L. Runnings, "An experimental analysis of two bone tools from the Manis site, Sequim, Washington," thesis, Washington State University, Pullman, WA (1984).

16. M. R. Waters, T. W. Stafford, Jr., H. G. McDonald, C. Gustafson, M. Rasmussen, E. Cappellini, J. V. Olsen, D. Szklarczyk, L. J. Jensen, M. T. P. Gilbert, E. Willerslev, Pre-Clovis mastodon hunting 13,800 years ago at the Manis Site, Washington. *Science* **334**, 351–353 (2011).

17. P. J. Reimer, W. E. N. Austin, E. Bard, A. Bayliss, P. G. Blackwell, C. B. Ramsey, M. Butzin, H. Cheng, R. L. Edwards, M. Friedrich, P. M. Grootes, T. P. Guilderson, I. Hajdas, T. J. Heaton, A. G. Hogg, K. A. Hughen, B. Kromer, S. W. Manning, R. Muscheler, J. G. Palmer, C. Pearson, J. van der Plicht, R. W. Reimer, D. A. Richards, E. M. Scott, J. R. Southon, C. S. M. Turney, L. Wacker, F. Adolphi, U. Büntgen, M. Capano, S. M. Fahrni, A. Fogtmann-Schulz, R. Friedrich, P. Köhler, S. Kudsk, F. Miyake, J. Olsen, F. Reinig, M. Sakamoto, A. Sookdeo, S. Talamo, The IntCal20 northern hemisphere radiocarbon age calibration curve (0–55 cal kBP). *Radiocarbon* **62**, 725–757 (2020).

18. G. Haynes, The millennium before Clovis. *PaleoAmerica* **1**, 134–162 (2015).

19. G. Haynes, Taphonomy of the Inglewood mammoth (*Mammuthus columbi*) (Maryland, USA): Green-bone fracturing of fossil bones. *Quat. Int.* **445**, 171–183 (2017).

20. C. V. Haynes Jr., B. B. Huckell, The Manis Mastodon: An alternative interpretation. *PaleoAmerica* **2**, 189–191 (2016).

21. L. C. Junquerira, J. Carneiro, *Basic Histology* (Lange Medical Publications, ed. 4, 1983).

22. A. Oryan, S. Monazzah, A. Bigham-Sadegh, Bone injury and fracture healing biology. *Biomed. Environ. Sci.* **28**, 57–71 (2015).

23. S. J. Olsen, *Osteology for the Archaeologist: The American Mastodon and the Woolly Mammoth* (Papers of the Peabody Museum of Archaeology and Ethnology, Volume 56, Number 3, 1972).

24. W. D. Allmon, P. L. Nester, *Mastodon Paleobiology, Taphonomy, and Paleoenvironment in the Late Pleistocene of New York State: Studies on the Hyde Park, Chemung, and North Java Sites* (Paleontological Research Institution, Palaeontographica Americana, Number 61, 2008).

25. J. Shoshani, G. H. Marchant, Hyoid elements from Mammut and Mammuthus from three Pleistocene sites, New York, USA, in *Mastodon Paleobiology, Taphonomy, and Paleoenvironment in the Late Pleistocene of New York State: Studies on the Hyde Park, Chemung, and North Java Sites*, W. D. Allmon, P. L. Nester, Eds. (Paleontological Research Institution, Palaeontographica Americana, Number 61, 2008), pp. 85–110.

26. D. Mariappa, *Anatomy and Histology of the Indian Elephant* (Indira Publishing House, 1986).

27. M. Sutton, Envisioning a western Clovis ritual complex. *PaleoAmerica* **7**, 333–364. (2021).

28. J. J. Miszkiewicz, T. J. Stewart, R. Naseri, A. Sołtysiak, The lifestyles of Bronze Age Zagros highlanders at Deh Dumen, Iran: Insights from midshaft femur cross-sectional geometry and histology. *Archaeometry* **64**, 1270–1287 (2022).

29. J. Trapani, D. C. Fisher, Discriminating proboscidean taxa using features of the Schreger pattern in tusk dentin. *J. Archaeol. Sci.* **30**, 429–438 (2003).

30. R. Sukumar, *The Living Elephants: Evolutionary Ecology, Behavior, and Conservation* (Oxford Univ. Press, 2003).

31. J. H. Poole, C. J. Moss, Musth in the African elephant, *Loxodonta africana. Nature* **292**, 830–831 (1981).

32. I. O. Buss, *Elephant Life: Fifteen Years of High Population Density* (Iowa State Univ. Press, 1990).

33. D. C. Fisher, Taphonomy and paleobiology of the Hyde Park mastodon, in *Mastodon Paleobiology, Taphonomy, and Paleoenvironment in the Late Pleistocene of New York State: Studies on the Hyde Park, Chemung, and North Java Sites*, W. D. Allmon, P. L. Nester, Eds. (Paleontological Research Institution, Palaeontographica Americana, Number 61, 2008), pp. 197–289.

34. D. C. Fisher, Paleobiology and extinction of proboscideans in the Great Lakes Region of North America, in *American Megafaunal Extinctions at the End of the Pleistocene*, G. Haynes, Ed. (Springer, 2009), pp. 55–75.

35. K. M. Smith, D. C. Fisher, Sexual dimorphism of structures showing indeterminate growth: Tusks of American mastodons (*Mammut americanum*). *Paleobiology* **37**, 175–194 (2011).

36. J. J. Saunders, *Late Pleistocene vertebrates of the Western Ozark Highland, Missouri* (Illinois State Museum, 1977), Report of Investigations 33, 118 pp.

37. J. H. Miller, D. C. Fisher, B. E. Crowley, R. Secord, B. A. Konomi, Male mastodon landscape use changed with maturation (Late Pleistocene, North America). *Proc. Natl. Acad. Sci. U.S.A.* **119**, e2118329119 (2022).

38. P. L. Nester, L. D. Brown, N. G. Miller, The Hyde Park mastodon site, Dutchess County, New York: Stratigraphy and basin profile based on field observation and ground penetrating radar, in *Mastodon Paleobiology, Taphonomy, and Paleoenvironment in the Late Pleistocene of New York State: Studies on the Hyde Park, Chemung, and North Java Sites*, W. D. Allmon, P. L. Nester, Eds. (Paleontological Research Institution, Palaeontographica Americana, Number 61, 2008), pp. 135–142.

39. R. M. Ross, F. Allaby, C. S. Butler, E. Y. Butler, D. Gabreski, L. Paciulli, K. J. Gremillion, W. D. Allmon, The Hyde Park Mastodon matrix project, with particular reference to the mollusks and seeds, in *Mastodon Paleobiology, Taphonomy, and Paleoenvironment in the Late Pleistocene of New York State: Studies on the Hyde Park, Chemung, and North Java Sites*, W. D. Allmon, P. L. Nester, Eds. (Paleontological Research Institution, Palaeontographica Americana, Number 61, 2008), pp. 111–134.

40. S. Reber, T. Simmons, Interpreting injury mechanisms of blunt force trauma from butterfly fracture formation. *J. Forensic Sci.* **60**, 1401–1411 (2015).

41. A. M. Christensen, V. A. Smith, Rib butterfly fractures as a possible indicator of blast trauma. *J. Forensic Sci.* **58**, S15–S19 (2013).

42. G. A. Otis, *The Medical and Surgical History of the War of the Rebellion, Part II, Volume II* (U.S. Government Printing Office, 1876).

43. G. A. Otis, D. L. Huntington, *The Medical and Surgical History of the War of the Rebellion, Part III, Volume II* (U.S. Government Printing Office, 1883).

44. A. Ramasamy, A. M. Hill, S. Masouros, I. Gibb, A. M. J. Bull, J. C. Clasper, Blast-related fracture patterns: A forensic biomechanical approach. *J. R. Soc. Interface* **8**, 689–698 (2011).

45. L. P. Vieira, F. P. Costa, P. Negrão, N. Neves, E. L. Monteiro, M. R. da Silva, “Bone-shot fracture”—An unusual iliac wing fracture caused by a projectile of autologous bone fragment. A case report. *Trauma Case Rep.* **33**, 100456 (2021).
